# Investigation of the Foaming Morphology of Polypropylene Molded via Microcellular Injection Assisted by Water Vapor and Gas Counter Pressure

**DOI:** 10.3390/polym17050611

**Published:** 2025-02-25

**Authors:** Shia-Chung Chen, Chao-Yuan Gan, Yan-Jun Liu, Ching-Te Feng

**Affiliations:** 1 R&D Center for Smart Manufacturing, Chung Yuan Christian University, Taoyuan 32023, Taiwan; jimmykan410@gmail.com (C.-Y.G.);; 2 Department of Mechanical Engineering, Chung Yuan Christian University, Taoyuan 32023, Taiwan

**Keywords:** microcellular injection molding, supercritical fluid foaming agents, water-assisted foaming, foaming morphology, bubble coalescence, gas permeability, gas counter pressure

## Abstract

The microcellular injection molding (MuCell^®^) process, which uses supercritical fluid (SCF) as a foaming agent, is considered an important green molding solution to reduce product weight, molding energy, and cycle time and to improve the foam quality. However, maximizing the foaming density while keeping size uniformity in the foaming cell requires further attention. In this study, H_2_O and the SCF N_2_ were employed as cofoaming agents in the MuCell^®^ process of polypropylene (PP). Owing to the different critical points of N_2_ and H_2_O, bubble nucleation was expected to occur in interactive ways. Various process parameters were investigated, including the SCF N_2_ content, the moisture content adsorbed within the resin under targeted PP weight reductions of 30% and 40%, the melt and mold temperature conditions, and the gas counter pressure. The resulting foaming morphology was examined to evaluate the foam quality in terms of the foaming density and bubble size distribution. The bubble coalescence, particularly in the skin layer, was examined, and the associated gas permeability flow rate was measured. The results indicated that H_2_O-assisted foaming led to bubble coalescence and allowed for gas penetration in the direction of the part thickness direction, resulting in an overall increase in foaming density, particularly in the skin layer. Under high SCF N_2_ and H_2_O contents, the solid skin layer disappeared, regulating the gas permeability from one surface side to the other. Under the optimized process parameters, the gas permeability flow rate in the filter-like foaming PP material reached 300–450 mL/min. The application of gas counter pressure also helped increase the foam density and bubble coalescence, enhancing the gas permeability in the PP material to about 500 mL/min. These results demonstrate the potential application of microcellular injection molding using water as a cofoaming agent in moisture-release devices.

## 1. Introduction

In recent years, environmental awareness in the plastics industry has promoted the use of green molding technologies toward energy efficiency and environmental conservation. Among such technologies, supercritical microcellular foaming stands out because it not only reduces product weight, molding energy, and resource waste but also shortens production times while improving the foam quality.

Supercritical microcellular foaming injection molding (MuCell^®^) was developed in the 1980s by Dr. Suh and his research team at the Massachusetts Institute of Technology and subsequently acquired by Trexel Inc. (Wilmington, MA, USA) and integrated into the injection molding process to create supercritical microcellular foaming injection molding machines. Compared with traditional injection molding techniques, the MuCell^®^ technology offers superior material properties, including low thermal conductivity, fair tensile and compressive strength, stability under high temperature and pressure conditions, a low dielectric constant, and improved fatigue resistance, impact strength, and toughness. Recently, global initiatives promoting environmental protection and energy conservation have accelerated the worldwide adoption and development of this technology, which has found applications in automotive interiors, electronic materials, medical devices, and shoe soles, among others.

**Figure 1:**
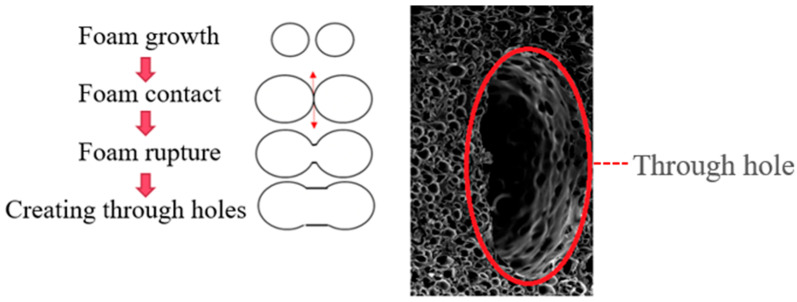
Schematic of the process of void formation due to bubble coalescence (adapted from [1]).

**Figure 2:**
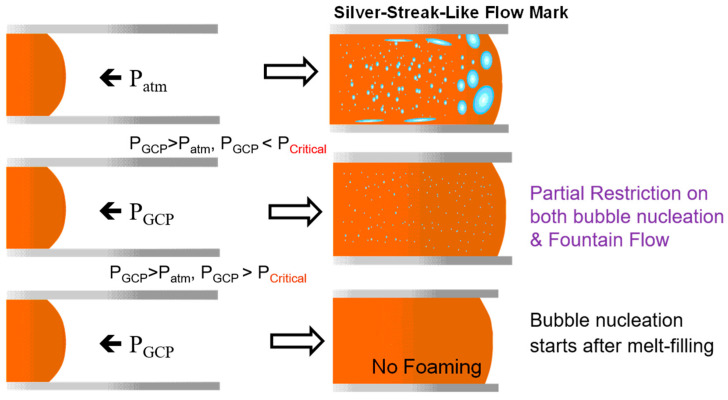
Schematic of the influence mechanism of the gas counter pressure (GCP) process on the microcellular injection molding process.

Dong and Zhao [1,2] found that during the filling stage, when the molten material passes through the gate, the front end of the melt experiences a pressure drop, which initiates the formation of bubble nuclei. Over time, these nuclei continue to grow into larger foam bodies or voids (Figure 1). The subsequent molten material passing through the gate undergoes a smaller pressure drop, resulting in less growth of foam bodies and thus the forming of smaller bubbles. This leads to the formation of bubbles with uneven sizes and densities within the foamed product. This phenomenon can be overcome using gas counter pressure (GCP). When the internal pressure of the mold cavity is greater than or equal to the critical pressure of the foaming agent, the foaming can be suppressed. By controlling the pressure with GCP, a uniform foaming effect can be achieved (Figure 2).

**Figure 3:**
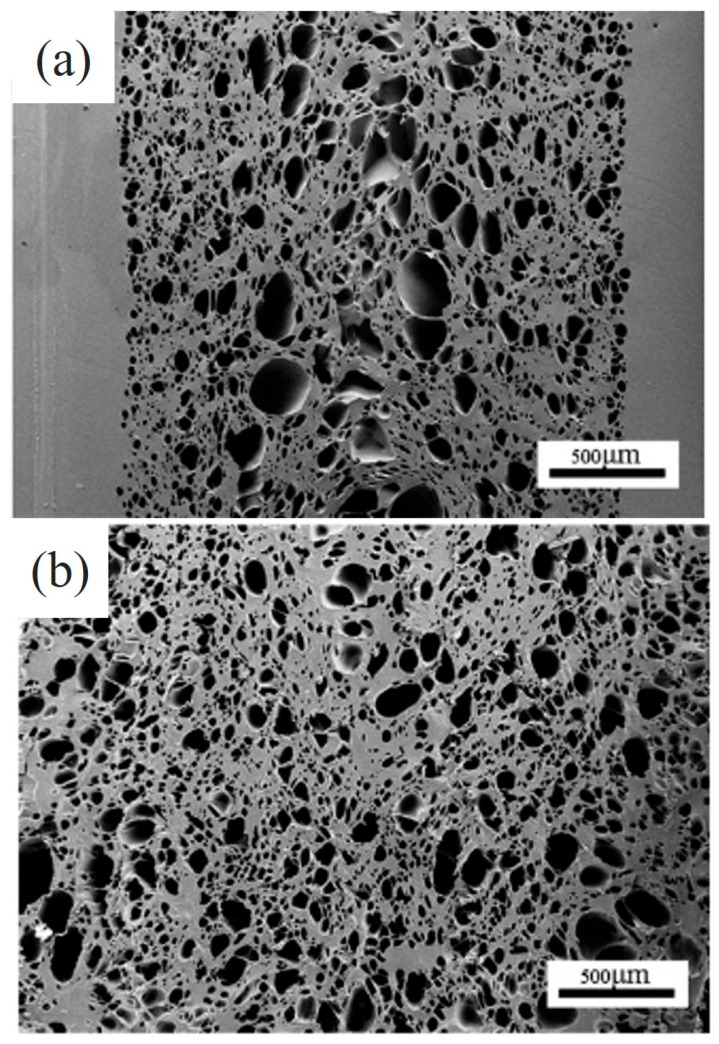
Schematic of the foam structure of TPU materials using CO_2_ and H_2_O. (**a**) Foaming and molding of TPU materials using CO_2_. (**b**) Cofoaming of TPU materials using CO_2_ and water. [3].

**Figure 4:**
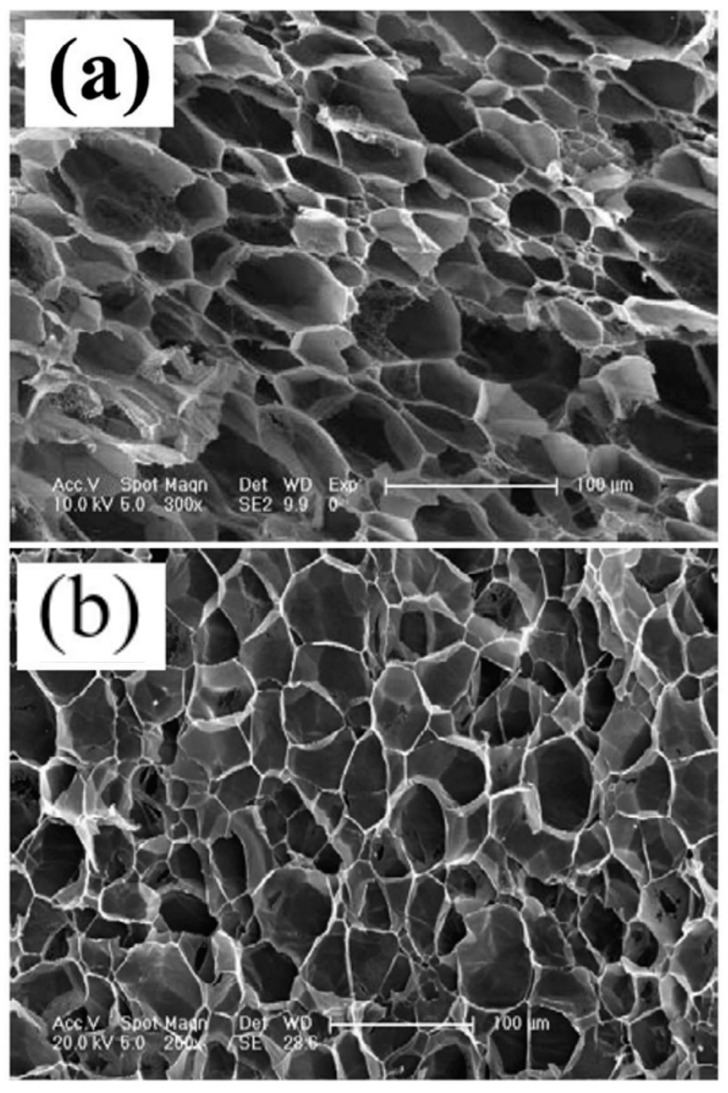
SEM images of the foam structure of PP with added hydrophilic materials using CO_2_. (**a**) Batch foaming of PP materials using CO_2_. (**b**) Batch foaming of PP materials with the addition of hydrophilic materials using CO_2_ [5].

Yang Mi et al. [3] used a thermoplastic polyurethane (TPU) material in a H_2_O-assisted foaming technology based on the microcellular injection molding (MIM) process using CO_2_ and H_2_O. Using H_2_O as a coblowing agent increased the sample porosity and effectively eliminated the skin layer (Figure 3). Ming Zhi Xu et al. [4] used polypropylene (PP) with H_2_O as the blowing agent for extrusion foaming, successfully producing samples with a high open-cell content. Minggang Li et al. [5] employed CO_2_ and H_2_O as foaming agents to prepare PP composite materials. These studies demonstrated that H_2_O plays a critical role as a physical foaming agent, enhancing the foam nucleation rate and ensuring a stable foaming extrusion while effectively controlling the foam density and structure (Figure 4).

The GCP technology [6,7,8] has been proven effective in controlling foam quality. GCP involves prefilling the mold with high-pressure gas before the filling stage and maintaining the pressure until the filling is complete. This delays the bubble nucleation during the filling stage, improving the surface quality. Chen et al. [9] confirmed that the GCP technology reduced surface defects, and that maintaining pressure for 10 s during GCP could prevent MIM from foaming until the molten polymer solidified.

In this study, the bubble coalescence between the core and skin layers during foam formation and its impact on the air permeability in the final products were investigated. A combination of weight reduction and cofoaming agents with the GCP technology is traditionally used to control the bubble size uniformity and minimize coalescence; however, this research aimed to achieve the opposite effect. This approach not only enhances the skin layer foaming rate but also promotes the bubble coalescence between layers, aiming to control the pore size and increase the air permeability toward applications in filtration devices. Table 1 provides an overview of research regarding the use of physical foaming and H_2_O-assisted foaming in PP materials, focusing on the cell size and cell density.

## 2. Experimental

### 2.1. MuCell^®^ Injection Molding Machine and GCP Regulation

An HT-150SV-MuCell^®^ FCS Taiwan equipped with a supercritical fluid (SCF) generator (T-100 Trexel, Wilmington, MA, USA) was used. The machine used N_2_ as the foaming agent. A homemade gas-pressure-regulating unit with a high-frequency gas control valve was used to provide the required GCP.

### 2.2. Experimental Mold

**Figure 5:**
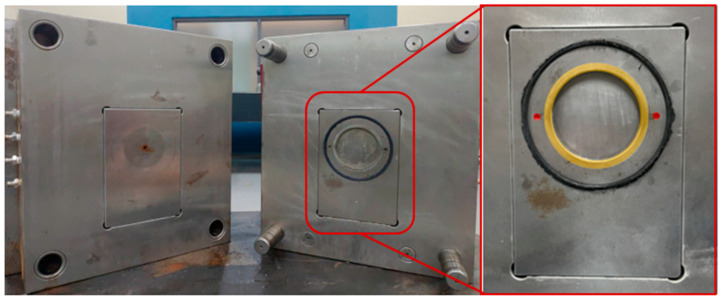
Mold design of a disc-shale mold used for the experiment. To apply the gas counter pressure, the mold needed a gas inlet (yellow color) and proper sealing (black O-ring).

**Figure 6:**
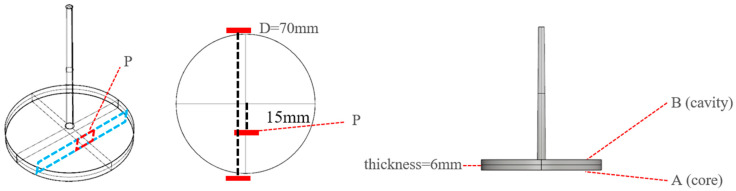
Schematic of the test sample locations where foaming morphology was measured. P was 15 mm away from disc center.

The mold was designed to allow high-pressure gas to fill the mold cavity, as shown in Figure 5. This mold design featured a sealing ring (black) around the mold core, and gas was introduced into the mold cavity through air vents (red) located on either side of the mold core. The inflation and exhaust were controlled using a GCP-controlling device. The pathway for the gas to enter and exit the mold cavity was designed as an exhaust channel (yellow) around the entire perimeter of the finished product, with a groove depth of 0.02 mm. The final dimensions of the mold were a diameter of 70 mm and a thickness of the circular plate of 6 mm. Figure 6 illustrates the measurement positions of the final product at different stages. The position of point P in Figure 6 is where the SEM images of the finished foam were taken for subsequent experimental measurements; A and B represent the skin layer orientation of the product corresponding to the foam SEM images in the subsequent experiments.

### 2.3. Materials

The material used in this study was PP-7533, produced by LCY CHEMICAL CORP Taiwan, which is an impact-resistant PP copolymer commonly used in automotive parts, electrical appliances, furniture, and battery cases. This material is a plastic elastic polymer with a processing temperature ranging from 190 °C to 270 °C.

### 2.4. Characterization

A moisture analyzer (MA37-1 Sartorius, Göttingen, Germany) was used to measure the moisture content (ppm) of the material before molding. The measurement method was thermogravimetric analysis.

The air permeability of the molded product was tested using an air permeability tester (TQD-G1 Labthink, Jinan, China). The testing was conducted according to the ISO 4638 [15] standard. Briefly, the air pressure was adjusted to create a constant pressure differential across both sides of the sample, and the air permeability flow rate of the gas passing vertically through the sample over a given area was measured within a specified time.

**Figure 7:**
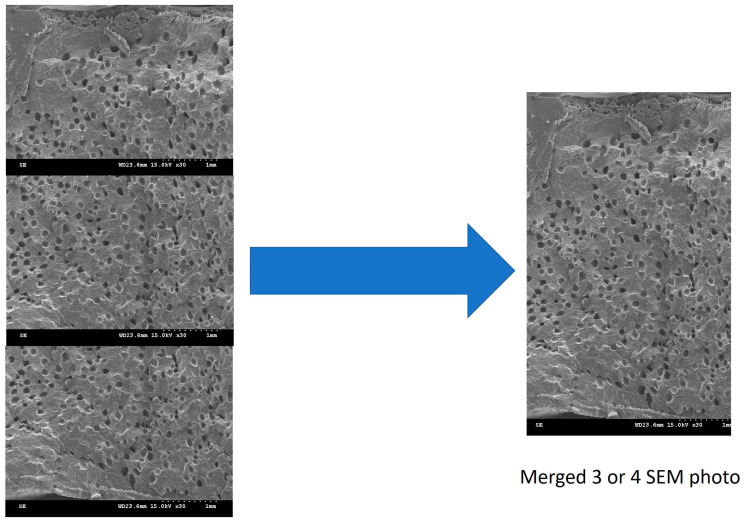
Schematic of SEM image merging.

The cellular structure of the sample was characterized using a scanning electron microscope (Model S-3000N Hitachi, Tokyo, Japan, 15 KV) and a metal coating unit (E-1010). Because the product thickness was greater than the height of a single image, the experimental SEM images of the product foam are presented as a composite of 3 to 4 images, as shown in Figure 7.

### 2.5. Experimental Parameters

First, the impact of the moisture content in the material on the MIM process was investigated. Under the fixed molding parameters shown in Table 2, experiments were conducted at various SCF contents, material moisture contents, and weight reductions of the final product. The specific parameters are listed in Table 3 (ID1-12). Next, GCP was applied to the molding process for the group with the optimal air permeability shown in Table 3 (ID13), and the effects on the sample skin thickness, average foam cell size (designated as numbers of cells per unit volume, in units of cells/cm^3^), foam density (defined as number of foam cells per unit cm^3^), and final product air permeability were observed.

## 3. Results and Discussion

### 3.1. Mucell Process

**Figure 8:**
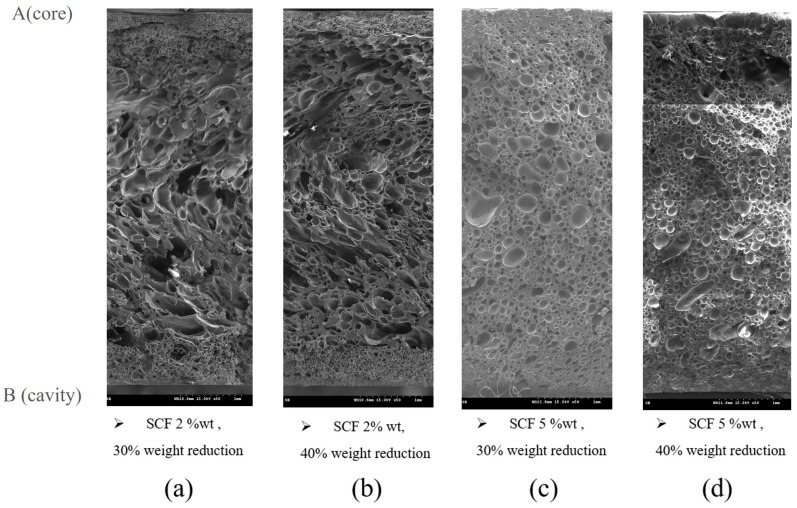
Foam morphology under different supercritical fluid (SCF) contents (without moisture content) and target weight reductions: (**a**) group 1 (2% SCF and 30% weight reduction), (**b**) group 4 (2% wt. SCF and 40% weight reduction), (**c**) group 7 (5% wt. SCF and 30% weight reduction), and (**d**) group 10 (5% wt. SCF and 40% weight reduction).

**Figure 9:**
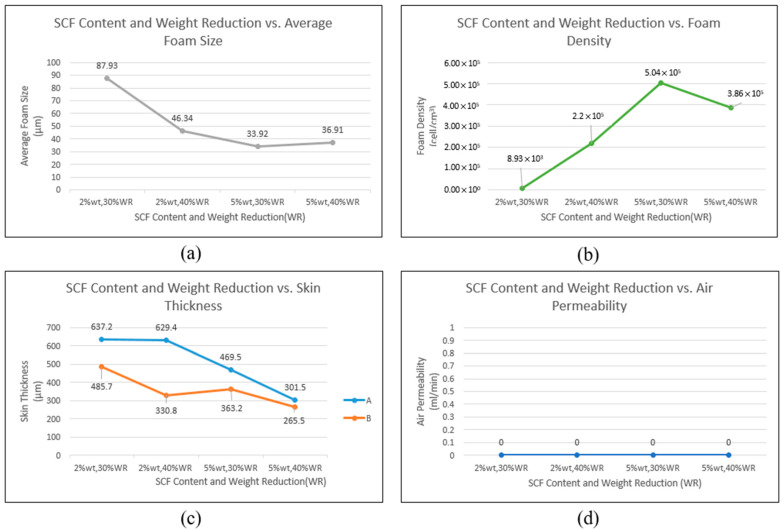
Influence of SCF content and target weight reduction on foaming qualities under moisture-free conditions. (**a**) Supercritical fluid (SCF) content and weight reduction vs. average foam size, (**b**) SCF content and weight reduction vs. foam density, (**c**) SCF content and weight reduction vs. skin thickness, and (**d**) SCF content and weight reduction vs. air permeability. The horizontal axis represents the corresponding four conditions for Groups 1, 4, 7, and 10.

Figure 8 shows the SEM images of PP without moisture (0 ppm), which display the foam morphology under different SCF contents and weight reductions. In particular, Figure 8a,b show the samples with 2 wt.% SCF content and weight reductions of 30% and 40%, respectively, and Figure 8c,d show the samples with 5 wt.% SCF content and weight reductions of 30% and 40%, respectively. Figure 9 presents the average foam size, foam density, skin layer thickness, and air permeability of these experimental groups.

A high SCF content led to a smaller average foam size and higher foam density. A similar trend was observed with increased weight reduction; however, at an SCF content of 5 wt.%, further weight reduction resulted in an opposite trend for the average foam size and foam density due to the occurrence of foam coalescence, as shown in Figure 9d. The skin layer thickness decreased with increasing SCF content and weight reduction, but the skin layer did not disappear; therefore, samples with air permeability could not be obtained. Table 4 summarizes the results for all experimental groups.

### 3.2. Mucell Assisted by H_2_O Adsorption and GCP

**Figure 10:**
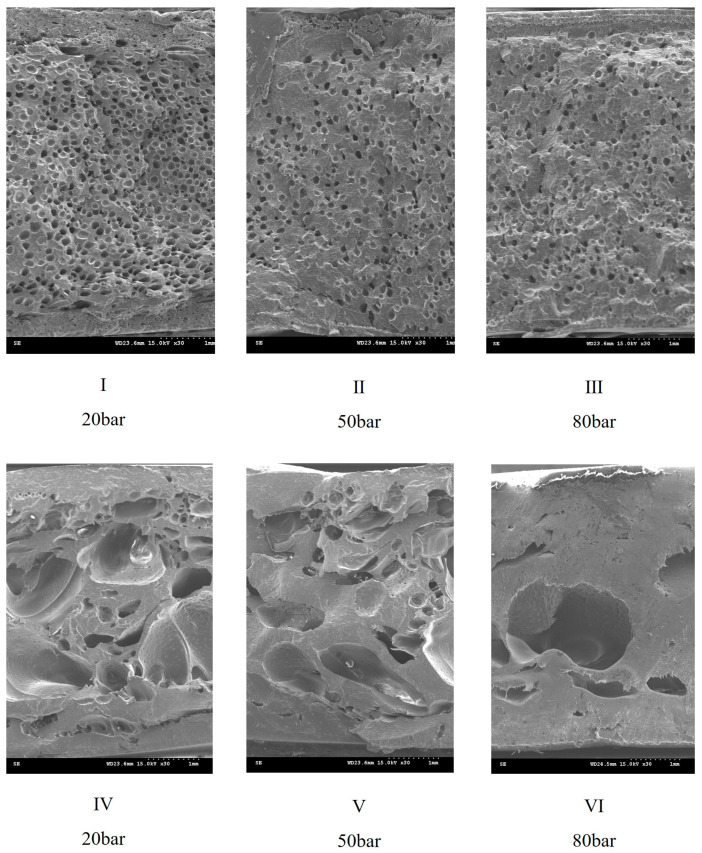
SEM images of N_2_ and H_2_O foaming at different gas counter pressures. (**I**–**VI** represent the molding conditions in Table 5).

As mentioned in the introduction, different foaming agents have varying saturation pressures. Experimental group 13 (Table 4) incorporated GCP to regulate the pressure within the mold cavity (Figure 1 and Figure 2), thereby controlling the timing of H_2_O and N_2_ foaming. This method ensured the uniform generation of more gas-filled pores, ultimately enhancing the gas permeability of the final product. Table 5 lists the experimental parameters for gas foaming at different pressures, and Figure 10 shows the corresponding SEM images. At a pressure of 80 bar (0.1 MPa), although the H_2_O and N_2_ foaming was not complete, a considerable decreasing trend was observed. Due to equipment limitations, 80 bar (0.1 MPa) was selected as the GCP for group 13.

**Figure 11:**
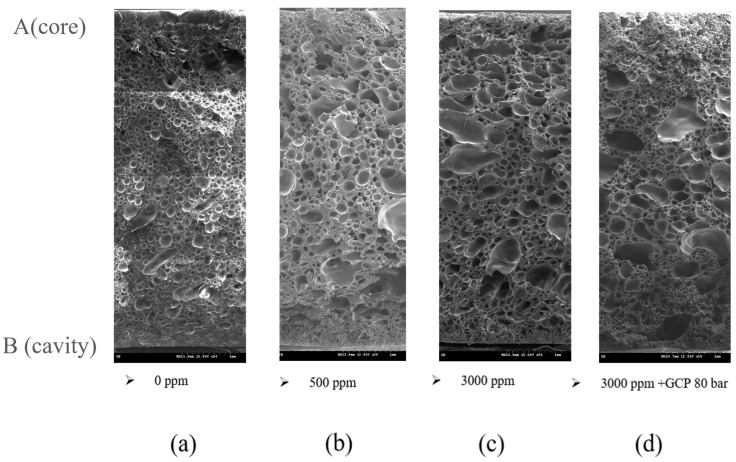
Foam structures obtained with 5% wt. supercritical fluid and 40% weight reduction, H_2_O-assisted foaming, and gas counter pressure (GCP): (**a**) group 10 (0 ppm moisture content), (**b**) group 11 (500 ppm moisture content), (**c**) group 11 (3000 ppm moisture content), and (**d**) group 13 (3000 ppm moisture content with a GCP of 80 bar (0.8 MPa)).

**Figure 12:**
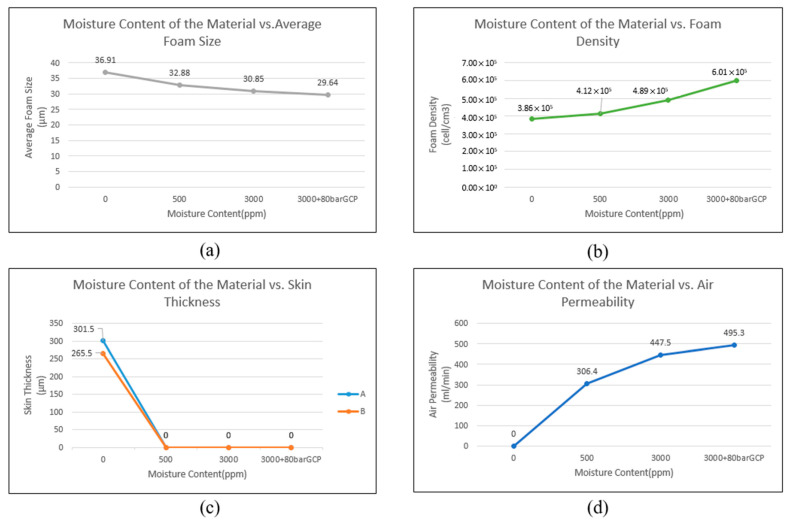
Effect of variations in moisture content on various foaming-related qualities under processing conditions of groups 10, 11, 12, and 13. (**a**) Average foam size, (**b**) foam density, (**c**) skin thickness, and (**d**) air permeability.

Figure 11 shows the foam structure for groups 10–13 in Table 3. Figure 11a–c depict the states of different material moisture levels under the same SCF content and weight reduction conditions, and Figure 11d shows the result of adding GCP at 80 bar (0.8 MPa) under the same SCF content, weight reduction, and material moisture conditions as those used in group 12. Figure 12 presents the average foam size, foam density, skin layer thickness, and air permeability of these experimental groups.

As the moisture content of the material increased, the foam size decreased and the foam density increased, indicating that H_2_O could act as a foaming agent in this experiment. The skin layer thickness also decreased with increasing moisture content until disappearing, which is consistent with previously reported trends. Finally, the air permeability of the samples also increased with increasing moisture content, demonstrating the potential of this method for producing breathable products. The results obtained for group 13, in which the GCP technology was used to control the foam growth for achieving a better foam structure, indicated that the GCP technology was beneficial for obtaining smaller foam sizes, higher densities, and, importantly, increased air permeability without a skin layer.

## 4. Conclusions

The results of using H_2_O and the SCF N_2_ as cofoaming agents in the MuCell^®^ process of PP can be summarized as follows:

In the filling stage of the MIM process, the blowing agent began to nucleate and foam due to a pressure drop. As the foam bubbles grew freely, they suppressed each other’s growth. Although there were still differences in the bubble size, which led to coalescence, the low foam density and high skin thickness prevented the final product from being permeable.

Increasing the SCF content resulted in an increase in the degree of foaming in the skin due to the increased number of foam bubbles, thereby reducing the skin thickness. In addition, the overall foam bubble size decreased as the number of nucleations increased, and the foam cells suppressed each other’s growth, resulting in a higher foam density. The decrease in skin thickness and the increase in nucleation number promoted the coalescence of foam bubbles, which helped connect the core-layer foam bubbles with the foam bubbles in the skin on both sides, forming through-holes that improved the air permeability.

When the moisture content in the material increased, the degree of foaming in the skin improved considerably, similar to the effect of increasing the SCF content. This was because the moisture in the material evaporated into steam at high temperatures, which effectively increased the gas content. The increased gas content helped enhance the foaming rate, leading to a higher number of foam bubbles and a reduction in skin thickness. Moreover, the foam bubble size decreased and the foam density increased as the moisture content increased.

As the weight reduction increased, the skin thickness decreased. Simultaneously, reducing the melt volume at the same gas content increased the foaming space in the final product, leading to larger overall foam bubble sizes and a decrease in foam density. When both the degree of skin foaming and the foam bubble size were enhanced, the coalescence of foam bubbles was promoted, which led to the formation of through-holes with foam bubbles on both sides of the skin, thereby improving the air permeability of the final product.

Using the GCP technology helped enhance foam nucleation. When H_2_O and N_2_, as cofoaming agents with different saturation pressures, were used, the GCP control caused the N_2_ used for foaming to dissolve back into the melt when the pressure exceeded the saturation pressure, whereas H_2_O was not fully dissolved back into the melt. By releasing the pressure, both foaming agents started to nucleate and grow. The difference in the foaming abilities of foam bubbles with different sizes enhanced their coalescence, leading to the formation of through-holes with foam bubbles on both sides of the skin. The results showed that under the testing condition of 1.72 bar (0.1 MPa), the air permeability increased by approximately 146%, demonstrating that the GCP control increased the air permeability compared with the condition without GCP control.

## Figures and Tables

**Table 1:** Foam properties of various materials and processes.

Research	Blowing Agent	Material	Advanced Features	Processing	Foam Size (µm)	Foam Density (cells/cm^3^)
Mi et al. [3]	CO_2_	TPU	H_2_O coblowing	Injection molding	135.4	1.9 × 10^5^
Xu et al. [4]	H_2_O	PP		Extrusion molding	200–1800	3 × 10^2^–1.82 × 10^5^
Li et al. [5]	CO_2_	PP	H_2_O coblowing	Batch foaming	17.3–36	3.7 × 10^8^–7.5 × 10^9^
Zhao et al. [10]	CO_2_	PVOH	H_2_O coblowing	Extrusion molding	8–25	1.25 × 10^10^
Kaewmesri et al. [11]	CO_2_	PP		Injection molding	3–8	5 × 10^8^–1 × 10^10^
Ishikawa et al. [12]	N_2_, CO_2_	PP	Core-back	Injection molding	100–400	1 × 10^11^–2.7 × 10^11^
Wang et al. [13]	N_2_, CO_2_	PP	RIC-FIM II	Injection molding	N_2_: 15–30 CO_2_: 15–20	N_2_:3 × 10^7^–3 × 10^8^ CO_2_: 7 × 10^7^–3.5 × 10^8^
Wang et al. [14]	N_2_, CO_2_, Ar, He	PP	Low pressure, core-back	Injection molding	N_2_: 28 CO_2_: 14 Ar: 18 He: 32	N_2_: 6 × 10^7^–8 × 10^7^ CO_2_: 4 × 10^8^–7 × 10^8^

**Table 2:** Fixed processing conditions for the MuCell^®^ injection molding.

Parameters	Value
Material	PP (LCY-7533)
Injection speed (mm/s)	70
Mold temperature (°C)	70
Material temperature (°C)	250
Back pressure (MPa)	4.9
Cooling time (s)	120

**Table 3:** Processing conditions for MuCell^®^ injection molding without (ID 1–12) and with GCP (ID 13).

Group ID	SCF Dosage (wt.%)	Weight Reduction (%)	Moisture Content (ppm)	GCP (Bar or 0.1 MPa)
1	2	30	0 (no moisture)	Non-applying
2	2	30	500	Non-applying
3	2	30	3000	Non-applying
4	2	40	0 (no moisture)	Non-applying
5	2	40	500	Non-applying
6	2	40	3000	Non-applying
7	5	30	0 (no moisture)	Non-applying
8	5	30	500	Non-applying
9	5	30	3000	Non-applying
10	5	40	0 (no moisture)	Non-applying
11	5	40	500	Non-applying
12	5	40	3000	Non-applying
13 (GCP)	5	40	3000	80

**Table 4:** Results of foam size, foam density, skin layer thickness, and air permeability for experimental groups 1–13.

Group	Average Foam Size (μm)	Foam Density (Cell/cm^3^)	Solidified Skin Thickness Core/Cavity (μm)	Air Permeability (mL/min)
1	87.93	8.93 × 10^3^	637.2/485.7	0
2	53.65	1.29 × 10^5^	638/481.9	0
3	32.19	3.73 × 10^5^	626/207.7	0
4	46.34	2.20 × 10^5^	629.4/30.8	0
5	42.26	2.95 × 10^5^	461.7/312.5	0
6	33.03	4.15 × 10^5^	307.4/145.7	0
7	36.91	5.04 × 10^5^	469.5/363.2	0
8	36.43	5.16 × 10^5^	259.5/203.4	0
9	35.19	5.37 × 10^5^	0/0	206.8
10	33.92	3.86 × 10^5^	3.1.5/265.5	0
11	32.88	4.12 × 10^5^	0/0	306.4
12	30.85	4.89 × 10^5^	0/0	447.5
13 (GCP)	29.64	6.01 × 10^5^	0/0	495.3

**Table 5:** Experimental parameters to evaluate the influence of the gas counter pressure (GCP) on the saturation pressure of the foaming agents.

Group	Foaming Agent	SCF Content (wt.%)	Moisture Content (ppm)	Weight Reduction (%)	GCP Pressure (Bar or 0.1 MPa)	Holding Time (s)	Cooling Time (s)
I	N_2_	5	0 (no moisture)	40	20	200	200
II	N_2_	5	0 (no moisture)	40	50	200	200
III	N_2_	5	0 (no moisture)	40	80	200	200
IV	H_2_O	0 (no additives)	3000	40	20	200	200
V	H_2_O	0 (no additives)	3000	40	50	200	200
VI	H_2_O	0 (no additives)	3000	40	80	200	200

## Data Availability

The original contributions presented in this study are included in the article. Further inquiries can be directed to the corresponding author.
